# Who’s afraid of Dura Mater?

**DOI:** 10.1016/j.bjorl.2024.101554

**Published:** 2025-02-06

**Authors:** Mario Emilio Zernotti, Javier Gavilán

**Affiliations:** a Sanatorio Allende, Department of Otorhinolaryngology, Córdoba, Argentina; b La Paz University Hospital, Madrid, Spain

**Keywords:** Bonebridge, Retrosigmoid, Middle fossa

## Abstract

**Objectives:**

To overcome the fear associated with conventional nomenclature related to different anatomic positions for Bonebridge.

**Methods:**

Bonebridge is an active transcutaneous semi-implantable bone conduction device available for clinical use since 2012. Bonebridge is indicated in patients with conductive and mild mixed hearing loss and a bone conduction threshold above 45 dB. This situation can be mainly found in patients with aural atresia, radical cavities, chronic otitis media and selected cases of otosclerosis not suitable for stapedectomy or hearing aids. The initial proposal was to position the implant in the mastoid, adjacent to the external ear canal. This position cannot be used in patients with radical cavities and should not be used in patients with aural atresia to avoid complications of future ear reconstructive surgery. New positions were soon implemented in the retrosigmoid and the middle fossa areas. However, the terms “retrosigmoid” and “middle fossa” are clearly linked to skull base surgery and create reluctancy among a large number of potential users.

**Results:**

To avoid the negative reaction of otologic surgeons to these names we propose the use of easy to remember names related to the anatomic location of the implant. These names have been presented to the scientific community in meetings over the last decade and are now published in this short paper.

**Conclusion:**

The new nomenclature proposed for the different anatomic positions available for Bonebridge is designed to stop the association between Bonebridge and skull base surgery.

**Level of evidence:**

Level 2.

## Highlights

• New names for Bonebridge positioning.
• Avoid “retrosigmoid” and “middle fossa”.
• Names include easy to remember acronyms of anatomic location.
• STELLA, PIA & MARA.

## Introduction

The first Bonebridge was implanted in 2011 as a part of a clinical trial.^1^ In 2012 the European controlled entry market of this new bone conduction device took place on a hotel hall at the airport of Munich. This was the official appearance of Bonebridge in the otologic community. Until that moment the percutaneous BAHA was the only device in the surgical bone conduction scenario, with the exception of some unsuccessful appearances like the passive transcutaneous Audiant^2^ which was discontinued due to problems related to the excessive pressure of the magnet on the skin.^3^

## Methods

Bonebridge started the era of active transcutaneous devices. Unlike their counterpart –percutaneous devices – Bonebridge was able to stimulate the inner ear via bone conduction while keeping an intact skin. The difference with passive transcutaneous devices was the lack of skin attenuation with Bonebridge since the new device was directly stimulating the bone.

**Fig. 1:**
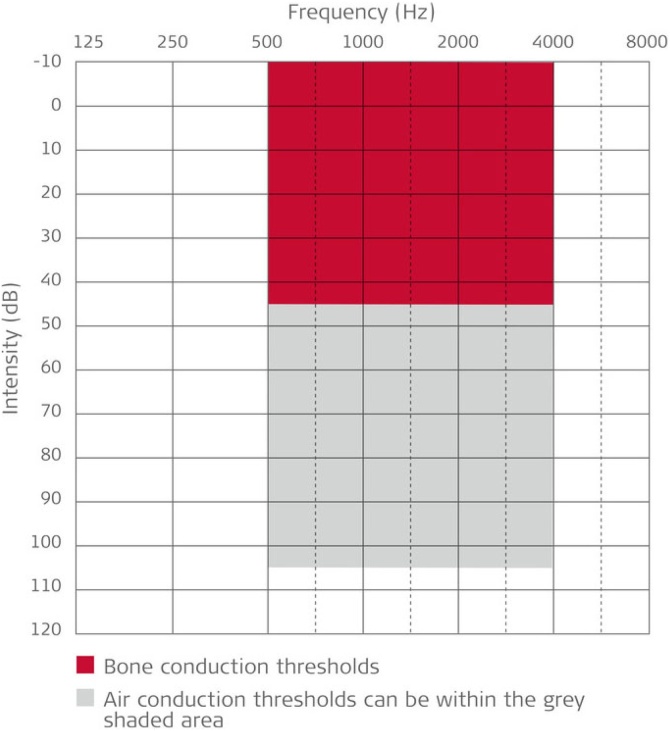
Bone conduction thresholds for Bonebridge.

Indications for Bonebridge included patients with conductive hearing loss with a bone conduction threshold up to 40–45 dB (Fig. 1). This situation can be encountered in congenital malformations – such as aural atresia – chronic ear disease, patients with radical cavities, and selected cases of otosclerosis, not amenable to stapedectomy due to medical conditions or surgical contraindications. Although Bonebridge official CE approval is for patients over 5-years of age, it has even been suggested that it can also be successfully implanted in children under 5-years.^4^, ^5^, ^6^

## Results

The initial proposal was to position the Floating Mass Transducer (FMT) – the vibrating part of the implant – in the mastoid, adjacent to the external ear canal.

Soon we discovered that the new device was a useful tool in our surgical armamentarium, with its main application in cases of aural atresia, and also patients with radical cavities suffering from a conductive or early mixed hearing loss.

The problem with these two indications is the same: the mastoid position of Bonebridge is not an option in these patients.

For patients with aural atresia, using the mastoid for Bonebridge may interfere future pinna reconstructive surgery. In cases with radical cavities, the reason was more obvious: no mastoid was available after the previous interventions.

Thus, to be able to use the new device to benefit patients in these groups, new positions needed to be implemented.

Our experience with the retrosigmoid approach in skull base surgery triggered the proposal of using the posterior placement of the FMT. The first retrosigmoid implantation of Bonebridge was performed by the authors (JG) on June 15, 2012, in a patient with a radical cavity at La Paz University Hospital in Madrid, Spain. This particular technique was published in 2013^7^ and has been used in most patients at that institution until the proposal of the middle fossa as an alternative to the mastoid position.

**Fig. 2:**
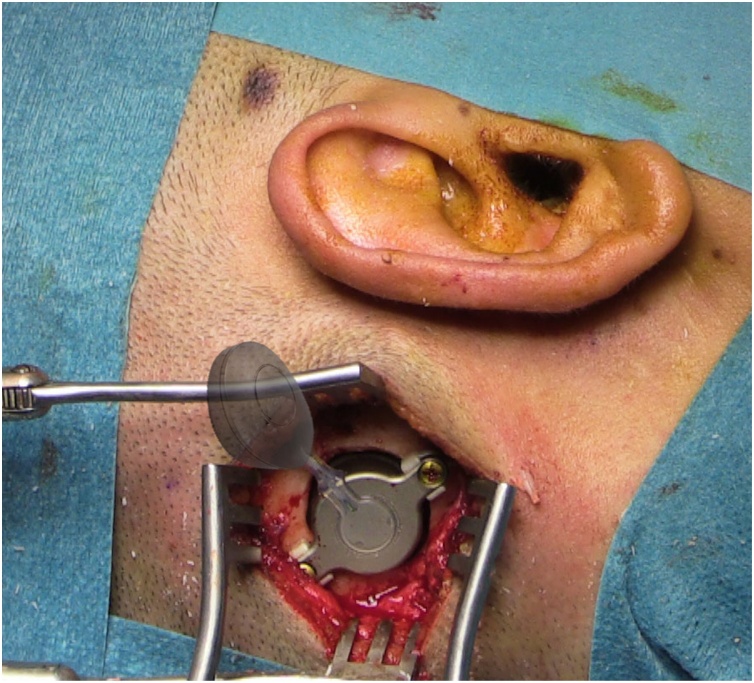
Retrosigmoid position for Bonebridge, with the approximate location of the speech processor.

The retrosigmoid position provides an acceptable placement of the FMT, allowing a proper position of the speech processor (Fig. 2).

**Fig. 3:**
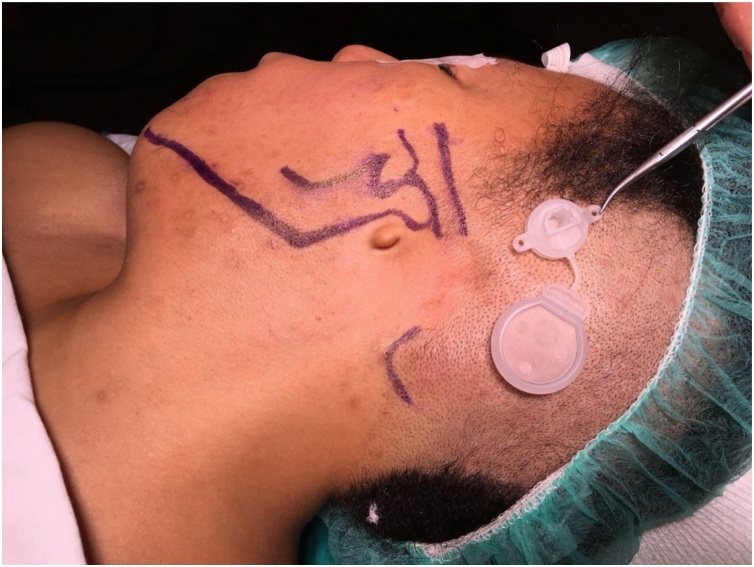
Middle fossa position for Bonebridge in an adult patient with aural atresia.

Something similar happened with the middle fossa position as an alternative to the “official” mastoid placement of the implant. It was first published by the authors (MEZ) in 2014^8^ and was soon accepted as a very good option for patients in whom the mastoid placement was not possible^9^ (Fig. 3). A new inverted middle fossa position has also been proposed to improve speech processor location.^10^

## Discussion

However, the terms “retrosigmoid” and “middle fossa” are clearly linked to skull base surgery and create reluctancy among a large number of potential users. Otologic surgeons without skull base surgery experience – in fact, the majority of potential Bonebridge users – do not like the idea of having to deal with structures like the dura mater and the sigmoid sinus for the placement of a “simple implantable hearing aid”. And, unfortunately, a large majority of potential candidates are in need of an alternative position due to anatomic reasons – atresia and radical cavities.

**Fig. 4:**
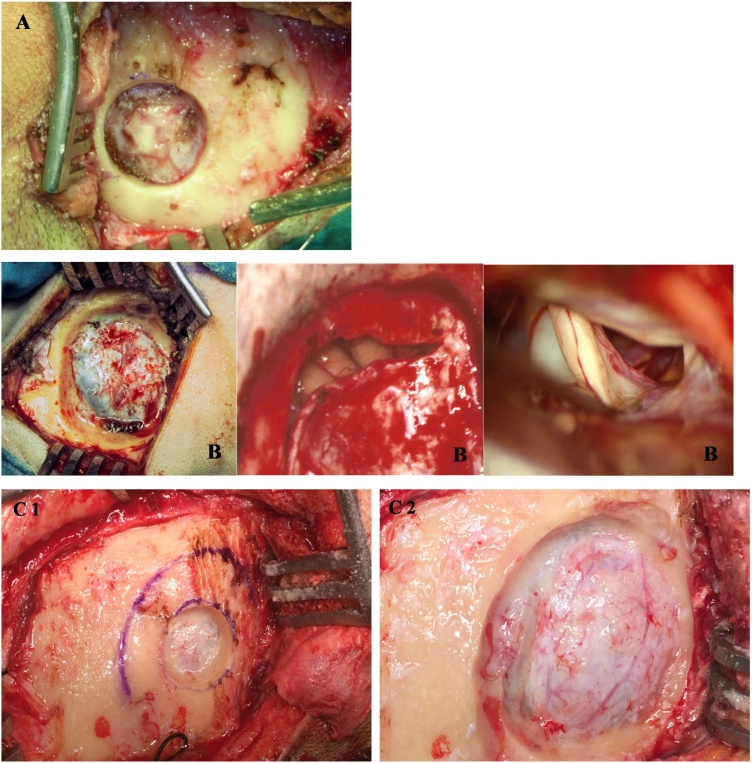
Comparison between a retrosigmoid craniotomy for Bonebridge and for vestibular neurectomy. (A) Retrosigmoid craniotomy for Bonebridge. (B) Retrosigmoid approach for vestibular neurectomy (B). (C) Size difference between the craniotomy for Bonebridge (1) and the craniotomy required for vestibular schwannoma removal (2).

**Fig. 5:**
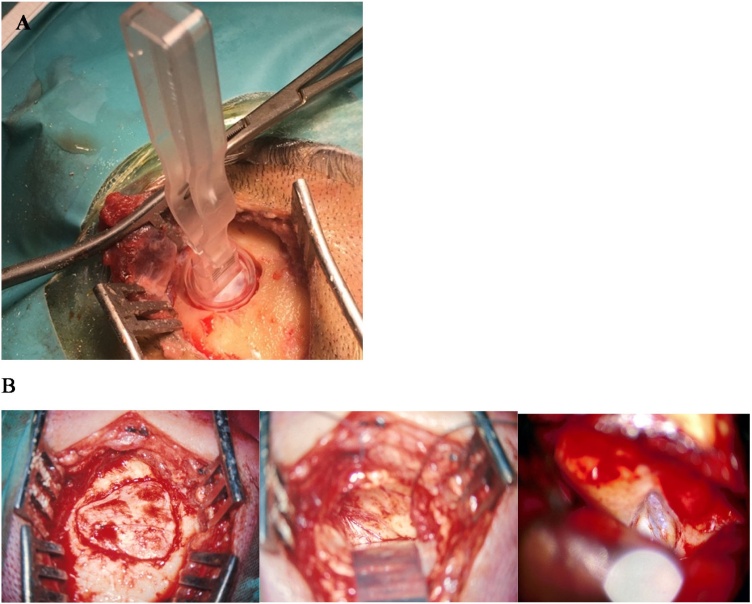
Comparison between a middle fossa craniotomy for Bonebridge (A) with a real middle fossa approach for a vestibular neurectomy (B).

Let’s be realistic. Bonebridge in the retrosigmoid area has nothing to do with a real retrosigmoid approach. A real retrosigmoid approach as shown in Fig. 4 includes a much larger craniotomy, opening of the posterior fossa dura and entering into the cerebello-pontine angle. The same happens with a real middle fossa approach (Fig. 5).

Since the position cannot be changed, why not change the name? And this is why we propose the use of simple female names to describe the anatomical options for the placement of the FMT. They are easy to remember since they have an anatomic link and do not carry the fear of vital structures associated with the procedure. The following names are proposed and, in fact, routinely used in our practice.

**Fig. 6:**
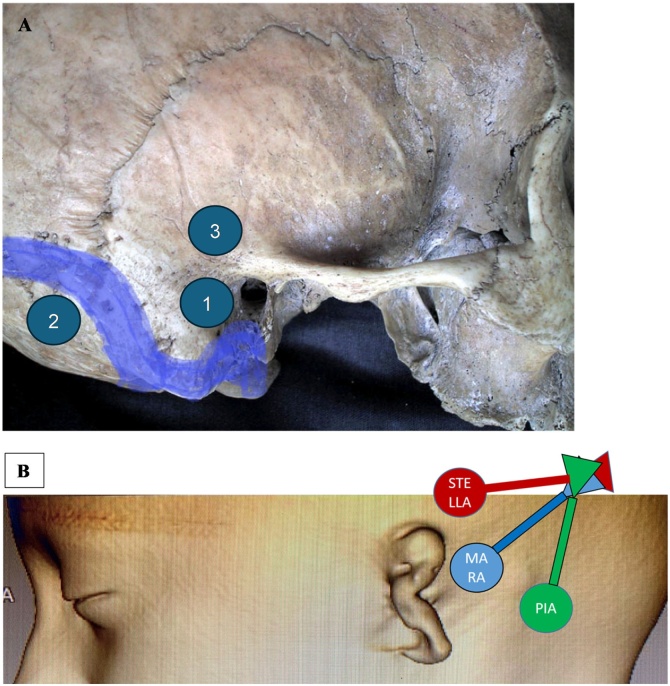
(A) Names without anatomic implications proposed to decrease the association of Bonebridge with skull base surgery. (1) MARA: Mastoid Regular Approach; (2) PIA: PosteroInferior Approach; (3) STELLA: SupratemporaL Line Approach. (B) The final position of the speech processor is the same regardless of the approach.

**MARA** was proposed to describe the Mastoid Regular Approach (conventional position). **STELLA** was selected for the **S**upra**TE**mpora**L L**ine **A**pproach (former middle fossa approach). And **PIA** was chosen to describe the **P**ostero-**I**nferior **A**pproach (former retrosigmoid approach) (Fig. 6).

However, changing names does not change the potential dangers associated with these positions. Is the alternative placement of Bonebridge in these areas really safe?

**Fig. 7:**
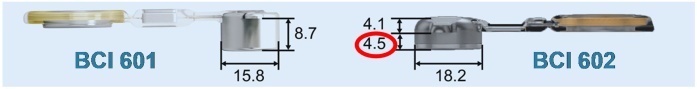
Differences in size and shape between first (BCI 601) and second (BCI 602) version of Bonebridge.

Our experience with PIA and STELLA supports the safety of these positions. Over the last 12-years, we did not experience any complication associated with the use of the implant in these areas. Furthermore, the long-term use of Bonebridge in the PIA and STELLA positions demonstrates the usefulness, not only of the implant itself, but also of the alternative placements.^11^ Also, headache is not an issue when considering the placement of the FMT in contact with the dura.^12^ This situation was more frequently needed with the previous version of Bonebridge (BCI 601) than with the new implant (BCI 602) due to the reduced depth of insertion with the current Bonebridge version: 4.5 mm in BCI 602 versus 8.7 mm with BCI 601 (Fig. 7).

Finally, concerning MRI in patients with Bonebridge, the STELLA position allows an artifact free visualization of the ipsilateral internal auditory canal, especially with the second version of Bonebridge – BCI 602 – when using metal artifact optimized sequences like MAVRIC.^13^

## Conclusion

Who’s afraid of Virginia Woolf is a 1966 American drama film directed by Mike Nichols. It stars Elizabeth Taylor, Richard Burton and George Segal. The film was nominated for 13 Academy Awards and won 5 Oscars. It is one of only two films to be nominated in every eligible category at the Academy Awards.

Of course, we do not intend to approach the geniality of these movie masters However, if these few lines help surgeons to increase the use of a widely recognized good solution for hearing loss in selected patients with conductive and early mixed hearing loss, we will be more than happy of our effort. No one should be “afraid” of the dura mater or the sigmoid sinus in the surgery of Bonebridge. These are structures that belong to the routine life of an otologist, who should learn to approach them with confidence and calm.

## Ethics committee

Ethics committee evaluation unnecessary because this is an opinion article without patients or animals involved in its production.

## Declaration of competing interest

The authors declare no have conflicts of interest.
